# Effects of Elevated Temperature and Food Supply on the Termination of Over-Summering and Subsequent Development of the Calanoid Copepod *Calanus sinicus*: Morphology, Physiology and Gene Expression

**DOI:** 10.1371/journal.pone.0161838

**Published:** 2016-09-21

**Authors:** Konglin Zhou, Minxiao Wang, Song Sun

**Affiliations:** 1 Key Laboratory of Marine Ecology and Environmental Sciences, Institute of Oceanology, Chinese Academy of Sciences, Qingdao, China; 2 University of Chinese Academy of Sciences, Beijing, China; 3 Laboratory for Marine Ecology and Environmental Science, Qingdao National Laboratory for Marine Science and Technology, Qingdao, China; 4 Jiaozhou Bay Marine Ecosystem Research Station, Institute of Oceanology, Chinese Academy of Sciences, Qingdao, China; University of Connecticut, UNITED STATES

## Abstract

The copepod *Calanus sinicus* Brodsky dominates the zooplankton in the Yellow Sea, China, and undergoes over-summering within the Yellow Sea Cold Water Mass (YSCWM). Termination of over-summering and subsequent development are regarded as key processes in population recruitment, and are probably linked to environmental variations in the YSCWM. In this study, we examined the effects of temperature (9 and 18°C) and food conditions (0.1 μg C mL^-1^ and unfed) on metabolic rates, morphological characteristics, and relative gene expressions of six genes involved in molting, gonad development, lipid catabolism, and stress tolerance processes of *C*. *sinicus* during termination of over-summering and subsequent development. Both elevated temperature and external food supply rapidly ended over-summering of *C*. *sinicus*, accompanied by up-regulation of the ecdysteroid receptor (EcR) gene expression and increased metabolic rates. These environmental conditions resulted in irreversible termination of over-summering and ensure the success of molting. During subsequent development, the lipid reserve in oil sacs could permit only early gonad development. The food supply might be a trigger to activate the final maturity of gonad by up-regulating expression of the vitellogenin receptor (VgR) gene. Thus, food played an indispensable role in population recruitment after termination of over-summering, whereas the elevated temperature accelerated these physiological processes. This study revealed the first dynamic profiles of physiological processes involved in over-summering termination and the subsequent development of *C*. *sinicus* using morphological, physiological and molecular methods simultaneously, confirmed the quiescent state of over-summering C5 copepodites, detected the effects of environmental changes on over-summering termination and subsequent development, and provided a foundation for future investigations of the mechanisms involved in over-summering in YSCWM.

## Introduction

In the pelagic ecosystems of the Yellow Sea, China, the copepod *Calanus sinicus* is the dominant species throughout the year and is the key intermediary to transfer primary production to higher trophic levels [1]. Although *C*. *sinicus* has a wide temperature tolerance range (5–23°C) [2, 3], a high surface temperature in summer (>26°C) has deleterious effects [4]. In summer, the population is dominated by C5 copepodites (C5s), and most individuals of *C*. *sinicus* undergo over-summering within the Yellow Sea Cold Water Mass (YSCWM, <10°C), which allows persistence of the population [5, 6]. The over-summering C5s are thought to be in a resting state, called quiescence [7, 8]. Unlike diapause, quiescence is a shallow form of dormancy that would suppress development during adverse conditions and in which development ensues immediately when environmental conditions improve [9]. The over-summering terminates when the YSCWM shrinks in autumn when the C5s molt into adults and contribute to the population recruitment in the Yellow Sea [10, 11]. But the specific processes controlling this over-summering are still unknown.

Similarly, other *Calanus* species (e.g. *C*. *finmarchicus*, *C*. *glacialis* and *C*. *hyperboreus*) undergo diapause in the North Atlantic and Arctic seas to survive in poor conditions (e.g. low food concentration and temperature) during winter [12–14]. Field studies of *Calanus* spp. have suggested the lipid hypothesis, which posits that diapause terminates when the copepods consume the lipid reserve to a certain degree [12, 15–17]. This hypothesis is supported by several population models [18, 19]. The seasonal decrease in oil sac sizes of *C*. *sinicus* also supports this hypothesis [11]. Furthermore, selective consumption of PUFA (polyunsaturated fatty acids) is thought to be a trigger of diapause termination of *C*. *finmarchicus* [20, 21].

Environmental factors (temperature, food-threshold concentration and photoperiod) have also been proposed as the triggers of termination of dormancy in the genus *Calanus* [22, 23]; however, no single factor can explain the termination of dormancy [24]. Strong vertical mixing of seawater in autumn changes the environmental conditions at the YSCWM edge, which might arouse over-summering C5s [10]. Increased temperature or elevated food conditions could promote the development of quiescent C5s [25].

The main characteristics of over-summering *C*. *sinicus* include low metabolic rates, suspended development, reduced diel vertical migration, a well-developed oil sac, and a low RNA:DNA ratio [7, 26, 27], as found in diapausing *C*. *finmarchicus* [28]. Ecdysis, gonad maturity, energy budget, and stress tolerance have been found to be important factors in dormancy in *Calanus* spp. [28, 29]. Elucidating the molecular control of these factors and their linkages to environmental conditons can improve overall understanding of the over-summering process and population dynamics. Our objectives were to: 1) identify the changes of physiological processes and associated gene expression levels during the termination and subsequent development; 2) link the gene expression patterns to the associated physiological processes; 3) understand the effects of environmental factors on the termination of over-summering and subsequent development; 4) explore the termination mechanism of over-summering for *C*. *sinicus*.

## Materials and Methods

We collected living *C*. *sinicus* from the central YSCWM. The over-summering C5s were cultured at two temperatures and two food conditions to test the effects of temperature and food supply on the termination and the subsequent development and maturity. Oxygen consumption rates (OCR) were measured to reflect variation in metabolic activity. The morphological characteristics of mandibular gnathobase and development stages of copepods in each group were monitored to study the molting process [30, 31]. Gonad development stages (GS) were recorded to explore the maturation process [32, 33], and the volume of the oils sac was used as a proxy of lipid content [34]. Molecular techniques [e.g. quantitative real-time PCR (qPCR) and high-throughput sequencing] were used to reveal more details of the physiological processes related to copepod dormancy by evaluating expression levels of associated putative genes [28, 35]. Recent transcriptome studies have provided valuable resources of gene sequences that are associated with internal processes in *C*. *sinicus* [36]. Thus, six target genes associated with molting, gonad development, lipid consumption and stress tolerance were measured by the qPCR method to detail their changes during the termination and subsequent development (Table 1).

**Table 1:** Genes and primer sequences of *Calanus sinicus* used in this study. GAPDH, EF1ɑ and 16S are housekeeping genes, while the other six are target genes.

Abbreviation	Gene name	Accession no	Primer for qPCR
GAPDH	Glyceraldehyde-3-phosphate dehydrogenase	KT947470	F:5'-ACTGACTTCTTGGGAGACACC-3'
			R:5'-TACCAAGAGATGAGCTTCACGAA-3'
EF1ɑ	Elongation factor-1-ɑ	KT947471	F:5'-GACAGGTCTCCAACGGAT-3'
			R:5'-CTTCTCCTTGATCTCAGCGAAC-3'
16S	16S	KT960998	F:5'-AATTAAATACTCCCGTGTG-3'
			R:5'-CAATCTGACTTACGTCGA-3'
EcR	Ecdysteroid receptor	KT947472	F:5'-CATGCCCTCAAAGAGCCTA-3'
			R:5'-ATTGCCAAGACTTCTCAGTTCG-3'
FAMeT	Farnesoic acid O methyltransferase	KT947473	F:5'-CAAGAACTGGATTCTGCGTCA-3'
			R:5'-ACTGTCTCTCCGTAGGCAC-3'
HOAD	Hydroxyacyl CoA dehydrogenase	KT947474	F:5'-GTCTCCACTTCTTCAACCCTGTCC-3'
			R:5'-TCAACAGTCATCTTCTTCATAGCCTT-3'
DI	Dienoyl-CoA isomerase	KT947475	F:5'-GATGACATTGCCAGGAAGTCCA-3'
			R:5'-CCAATCACTGGCTTCTTGCACT-3'
Ferritin	Ferritin	KT947476	F:5'-AACCGTGATGATCAAGCTC-3'
			R:5'-CGCTTGGTCTGATATTCCAT-3'
VgR	Vitellogenin receptor	KT947478	F:5'-TTTTCCAACAAGCTGAAGTCTCCC-3'
			R:5'-CCCAATGTTAGGCATGAAGTGGT-3'

### Sampling and preparation

**Fig 1:**
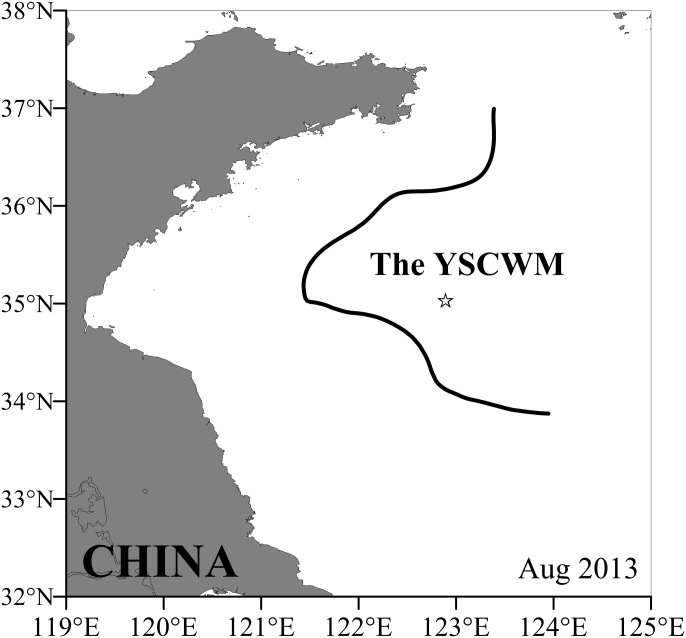
Sampling station for *Calanus sinicus*. The sampling station is marked with a star (☆) and is located inside the Yellow Sea Cold Water Mass **(**YSCWM). The boundary of the YSCWM is defined by the 10°C bottom isotherm and marked with bold black line.

Samples were collected within the YSCWM (122.89°E, 35.03°N) aboard the R. V. “Beidou” in the southern Yellow Sea, China from 16 Aug to 1 Sep 2013 (Fig 1). Live *C*. *sinicus* were hauled from 4 m above the bottom to 35 m depth (beneath the thermocline) using an 80-cm diameter closing net (mesh size: 330 μm) to ensure that only the over-summering population was collected. After sampling, the copepods were transferred immediately to a 20-L incubation barrel filled with pre-cooled bottom seawater (about 9°C). About 45 C5-stage copepods were picked out haphazardly with a wide-mouth pipette and kept temporarily in a 10-cm wide cylinder with pre-cooled (about 9°C) filtered seawater (FSW, filtered through a 0.45-μm pore size cellulose acetate filter) for the oxygen consumption rate measurements. Another 15 C5s were washed by the FSW and then three copepods each were put into five 1.5-mL frozen tubes to measure gene expression. The seawater was removed via bibulous paper. Copepods for RNA samples were flash frozen and kept in liquid nitrogen until analysis. An additional ~1,600 healthy C5s were selected haphazardly for the later culture experiment.

### Culture experiment

The C5s copepodids were cultured at two temperatures and two food conditions in two incubators (9°C and 18°C), including Group 9NF (9°C not fed), 9F (9°C fed), 18NF (18°C not fed) and 18F (18°C fed). The temperatures were typical for the YSCWM (9°C) and the middle of the thermocline (18°C). 18°C also was within the temperature range of tidal fronts when the YSCWM shrinks in autumn [10]. In groups 18NF and 18F, the copepods were kept at 9°C at first, and then the temperature was set 1°C higher every two hours until 18°C. In Groups 9F and 18F, food (the diatom *Skeletonema costatum* and the dinoflagellate *Prorocentrum micans*) was added every two days and the concentration was kept at about 0.1 μg C mL^-1^. For each group, ~400 C5s were kept in two 10-L plastic containers with FSW. The seawater was renewed every two days. Samples were taken after 1, 3, 6, 9, 16, and 31 days incubation to test the oxygen consumption rates and gene expression levels. 45 copepods for oxygen consumption rate measurements and 15 for gene expression analysis were sampled from each group. The data were marked as Day 1–31 (D1-31) in the results.

### Metabolic rates

The water-bottle method with brown glass bottles (about 500 mL) was used to ascertain the oxygen consumption rates. The bottles were filled with FSW at the culture temperatures by siphon to avoid creating bubbles. About 15 healthy copepods were transferred by a pipette into each bottle after gently washing. Three replicates were prepared for each treatment group and three bottles with only FSW were prepared simultaneously as controls. The bottles were sealed and kept at the culture temperatures in the incubators for 24–30 hours in dark.

After incubation, two ~130-mL water samples were siphoned from each bottle to determine the dissolved oxygen concentration based on the Winkler method [37]. The copepods were counted after the experiment and then preserved in 5% formalin seawater solution for later morphometric analysis. To eliminate the effect of body mass on metabolic rates, the carbon-weight-specific oxygen consumption rate (OCR, μL O_2_ mg^-1^ h^-1^) was calculated by the formula: $OCR\;=\;\frac{0.7\times (C_{0}-C)\times V}{n\times t\times CW}$, where 0.7 was a constant to transform 1 μg O_2_ into corresponding volume, C and C_0_ represented the oxygen concentration (mg L^-1^) in experimental and control bottles, respectively, V (mL) was the volume of incubation bottle, n was the number of copepods in incubation bottle, t (h) was incubation time, CW (mg) was mean carbon weight of copepods calculated from prosome length (PL) in the equation: lg(CW) = -9.416+3.378lg(PL) [38].

The lipid cost for respiration (R, μg) per copepod was estimated using OCR [26, 39]: $R\;=\;\sum \frac{OCR\times CW\times D\times 0.536RQ\times 24}{0.8}$, where OCR was the mean value, D represented the cultivation days, RQ (respiration quotient) was 0.7 for fat metabolism [40], 0.536 RQ was a factor to convert the respiration into carbon, and 0.8 was the carbon composition in lipid [39].

### Morphometrics

The lengths and widths of the prosome and oil sac of about 15 copepods in a sample were measured using a stereomicroscope (Zeiss Stemi SV11 Apro), three replicates for each group. The oil sac volume (OSV) was calculated by the formula: $OSV\;=\;\frac{\pi }{6}\times L\times W^{2}$ and the prosome volume (PV) was calculated by the equation: PV = 0.58 × L × W^2^ [34]. To remove the effect of body size, oil sac proportion (OSV%) was defined as the OSV/PV ratio to represent the relative amount of lipid reserve[11]. The lipid (μg) in the oil sac could be estimated from the oil sac volume (OSV): Lipid = OSV × ρ, where ρ was the lipid density (0.91 g mL^-1^) [29]. The developmental stages also were determined. The beginning of adult stage was defined as the time when 50% of the individuals had molted into adults [38]. Four gonad developmental stages of females also were determined (GS1, GS2, GS3 and GS4) [41]. Apolysis begins when the epidermis separates from the cuticle in mandibular gnathobase, which is a transition towards molting [31]. The mandibular gnathobases of C5s were extracted using a stereomicroscope and then observed and photographed using an Olympus BX51 differential-interference microscope. Jaw phases were assessed by the morphological characteristics of the mandibular gnathobase and were classified into pre-apolysis and post-apolysis [30, 31].

### Relative gene expression analysis

#### RNA extraction and cDNA synthesis

Total RNA was extracted from samples of three copepods with trizol (Invitrogen, Carlsbad, CA, USA), with five replicates from each treatment group. The quality and quantity of RNA was assessed using 1% agarose gel electrophoresis and Nanodrop 1000 (Thermo Fisher Scientific, Waltham, MA, USA). RNA samples with high quality (8 μL) were reverse-transcribed into cDNA using the RevertAid First Strand cDNA Synthesis Kit (Fermentas, Lithuania). The cDNA samples were stored at -20°C.

#### Quantitative real time RT-PCR (qPCR) and data analysis

The putative genes were chosen from the transciptome sequence of *C*. *sinicus* [36] based on mapping results against the NCBI non-redundant (Nr) protein database via BLASTX. The validity of the candidate genes have been confirmed by sequencing the PCR products amplified by the primers based on the corresponding unigenes in the *C*. *sinicus* transcriptome. QPCR Primers were designed based on the sequences obtained using Oligo 7 (Table 1).

The qPCR was performed on the Eppendorf Mastercycler (Eppendorf, Hamburg, Germany) using a DyNAmo Color Flash SYBR Green qPCR Kit (Thermo Fisher Scientific). The reactions were run in duplicate wells for each test, in a total volume of 20 μL containing 10 μL of Master mix, 1 μL each of the primers, 2 μL of cDNA, and 6 μL of RNase-free water. The qPCR programs followed the protocol: 95°C for 7 min, followed by 40 cycles at 95°C for 10 s and 60°C for 15 s. All the templates were diluted to 0.2 ng μL^-1^ with RNase-free water to ensure the Ct value was within the range of 18 to 30.

Expression of target genes was normalized to the geometric mean of three housekeeping genes EF1ɑ, 16S, GAPDH. Because the C5s within the YSCWM in summer were quiescent based on previous studies [6–8], the cDNA samples of C5s at the sampling station were chosen as the control group to calculate the relative gene expression for each target gene in the treatment groups. The data of C5s at the sampling station were marked as Day 0 (D0) in the results.

### Statistical analyses

All the statistical analyses were performed with SPSS 16.0 software. One-way ANOVA and multiple comparisons (LSD) were conducted to test for differences in morphological characteristics and gene expression levels. Relative gene expression data were log-transformed to obtain homogeneity of variance. The multiple comparisons were conducted and a significant difference was accepted when *P* < 0.05. Spearman correlation analysis was used to test for relationships between gene expression and morphometric data.

### Ethics statement

No vertebrates were sampled or used in our study. No specific permissions were required for the field studies. The studied area in the Yellow Sea is not privately owned or protected in any way. No endangered or protected species were involved either.

## Results and Discussion

**Fig 2:**
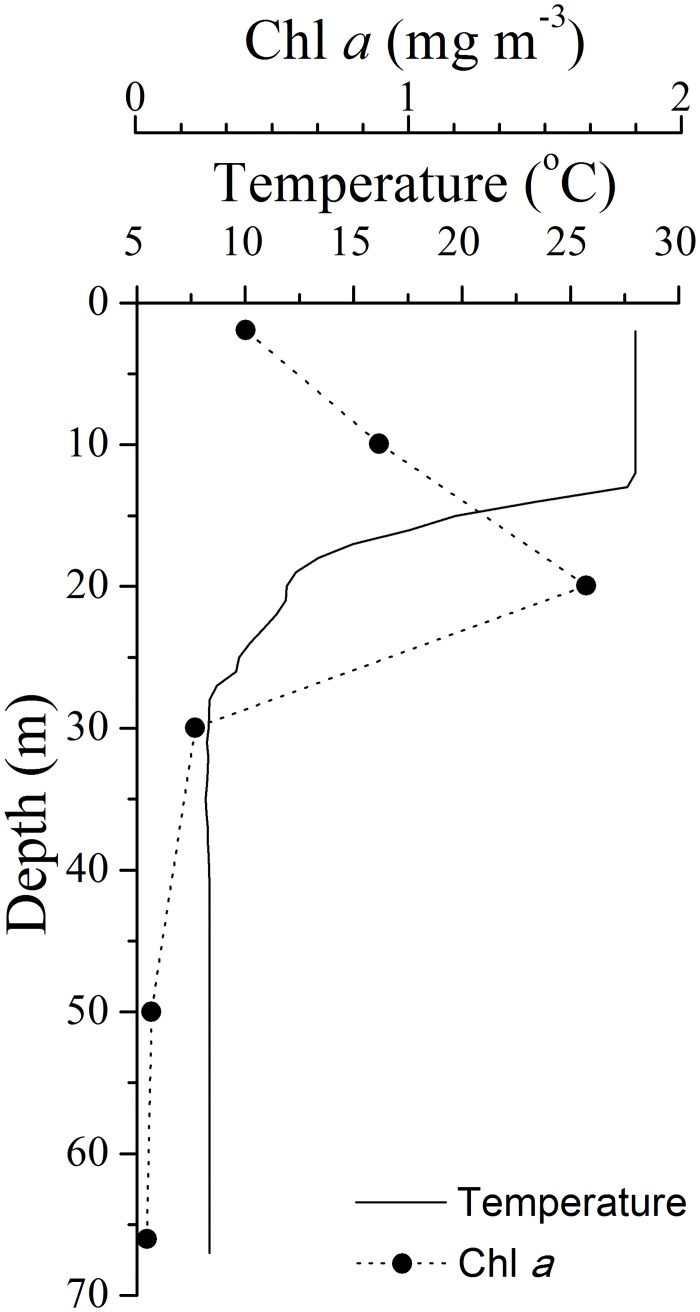
Vertical profiles of temperature and Chl *a* concentration at the *Calanus sinicus* sampling station.

The sampling station was located within the YSCWM (Fig 1), with the boundary defined by the 10°C bottom isotherm [42]. The sampling station had a strong thermal stratification between 10 and 30 m depth (Fig 2). The low bottom temperature (8.4°C) protected the *C*. *sinicus* from the high surface temperature (28°C), while the poor food conditions in bottom water (Chl *a* concentration: 0.1 mg m^-3^; Fig 2) limited the development of copepods [4]. The over-summering C5s had low metabolic rates (0.13 μL O_2_ ind.^-1^ h^-1^) and large oil sacs (OSV%: 0–30.67%), consistent with previous studies of the characteristics of the quiescent C5s within the YSCWM [7, 8, 11].

### OCR variation and over-summering termination

**Fig 3:**
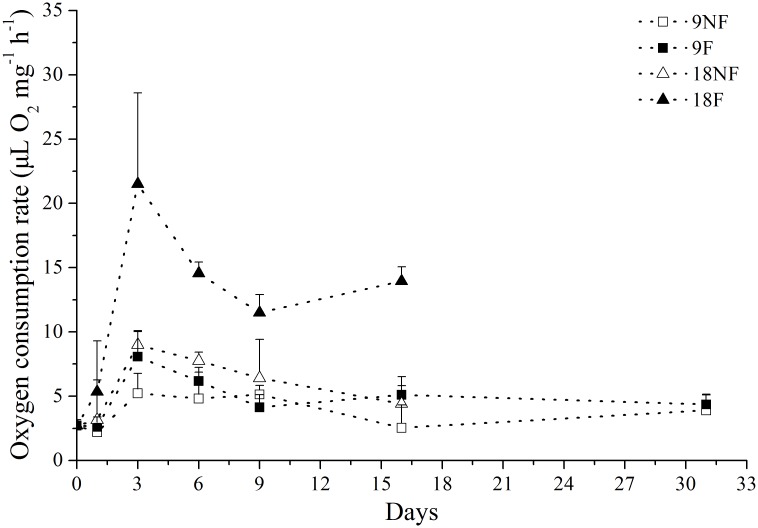
Oxygen consumption rate (OCR) of *Calanus sinicus* cultured under different temperatures by food environments. 9NF = 9°C not fed, 9F = 9°C fed, 18NF = 18°C not fed and 18F = 18°C fed.

The carbon-weight-specific respiration rates (OCR) varied significantly during the experiment under constant temperature cultivation (Fig 3), suggesting a wide fluctuation of metabolic activity in each group during the experiment. On Day 1, copepods in Group 18F had a slightly higher respiration rate than on Day 0, while the other groups did not. Copepods in all groups had significantly higher OCRs on Day 3 than on Day 0 (ANOVA, *p* < 0.05) and maintained high OCRs for several days, coincident with the energy-consuming molting process. The OCR in Group 18F was significantly higher than in the other three groups (ANOVA, *p* < 0.01), suggesting the combined effects of elevated temperature and food supply on copepods. Furthermore, on Day 16, the OCR increased again in both fed Groups 18F and 9F, consistent with the energy-consuming gonad development process.

The increased respiration rate on Day 3 might have indicated the termination of over-summering. A more rapid response was observed at higher temperature with a food supply. A time lag of 12 to 17 days, when endocrinological and biochemical processes towards molting occur, exists before diapausing *C*. *finmarchicus* starts the terminal molt when environment changes [12]. This preparatory period was much shorter in *C*. *sinicus*, suggesting a more rapid response to the environmental changes and stronger environmental adaptability. *C*. *sinicus* within the YSCWM should be quiescent and, when faced with either increased temperature or elevated food conditions, the metabolic activity is enhanced within three days. Nevertheless, C5s cultured without food at low temperature (Group 9NF) also showed an increased respiration rate on Day 3. Other factors also might have induced termination of quiescence of copepods in the experiment, such as stress due to hydrostatic pressure change, mechanical stimuli from sorting, and sudden exposure to light [43]. Thus, environmental changes could end the over-summering of *C*. *sinicus*.

### Molting development

**Fig 4:**
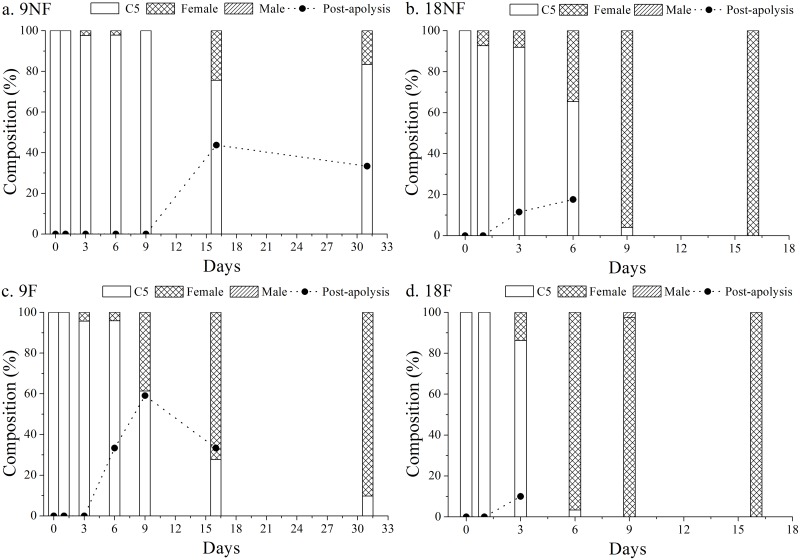
The composition time series of developmental stages and jaw phases in *Calanus sinicus*. (a) 9NF (9°C not fed), (b) 18NF (18°C not fed), (c) 9F (9°C fed) and (d) 18F (18°C fed).

C5s molted into adults successively in all the groups after termination (Fig 4). Copepods entered the adult stage after 4.3, 6.7 and 11.4 days in Groups 18F, 18NF and 9F, respectively; however, only 2–24% of C5s molted into adults in Group 9NF. Thus, higher temperature and better food conditions accelerated the molting process.

**Fig 5:**
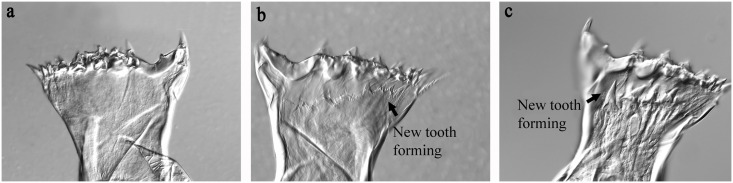
Jaw phase morphology of *Calanus sinicus*. (a) pre-apolysis on Day 0; (b) post-apolysis on Day 6 in Group 9F (9°C fed**)**, tooth forming; (c) post-apolysis on Day 9 in Group 9F. Scale bars: 20 μm.

**Fig 6:**
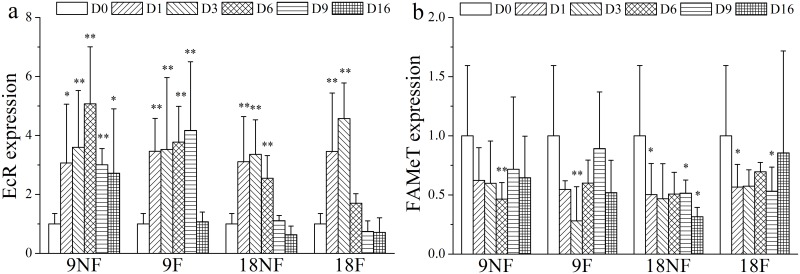
Relative gene expression variation of genes associated with the molting process of *Calanus sinicus*. (a) EcR (ecdysteroid receptor) expression. (b) FAMeT (Farnesoic acid O methyltransferase) expression. 9NF = 9°C not fed, 9F = 9°C fed, 18NF = 18°C not fed and 18F = 18°C fed. D0 to D16 represent Days 0 to 16. The expression level was normalized to the geometric mean of three housekeeping genes (GAPDH, 16s, EFα1). Error bars show the standard deviation. Significant differences (LSD): *P* < 0.05 (*); *P* < 0.01(**).

Apolysis started on Day 3 in Groups 18F and 18NF, on Day 6 in Group 9F, and on Day 16 in Group 9NF before the beginning of the adult stage, suggesting preparation for molting (Figs 4 and 5). The expression level of genes involved in molting process, however, had an earlier response. EcR (ecdysteroid receptor) expression was up-regulated and FAMeT (farnesoic acid O methyltransferase) was down-regulated significantly on Day 1 (Fig 6, ANOVA, *p* < 0.01). High expression of EcR lasted for 3, 6, 9 and 16 days in Groups 18F, 18NF, 9F and 9NF, respectively. Then the proportions of post-apolysis copepodids increased to maxima of 10, 17.6, 59.1 and 43.8% in Groups 18F, 18NF, 9F and 9NF, respectively, just before the beginning of the adult stage. Post-apolysis occurred after the up-regulation of EcR expression, which was similar to *C*. *finmarchicus* [31]. Thus, EcR expression should be a good molecular marker to indicate the molting process.

By binding to ecdysteroids, EcR could regulate molting [44]. The concentration of ecdysteroid increases before molting in crustaceans, as well as the relative expression of EcR [24, 45–47]. Diapausing *Calanus* copepods suppress the molting process and down-regulate EcR expression during the diapause period [28, 46]. Thus, the immediate up-regulation of EcR expression on Day 1 suggested the termination of quiescence and preparation for final molting, similar to diapause termination of *C*. *finmarchicus* [28, 48]. The EcR expression level was positively correlated with the proportion of C5s (Spearman correlation analysis, *r* = 0.537, *P* < 0.05). The down-regulation of EcR expression in the late incubation period could be related to negative feedback loops when most of the C5s molted into adults. On the other hand, FAMeT participates in methyl farnesoate (MF) biosynthesis [49], a juvenile hormone-like compound that is in low concentration to promote copepod adult maturation during the final molting [49–51]. The decreased expression of FAMeT here could be a response to the final molting.

### Gonad development

**Fig 7:**
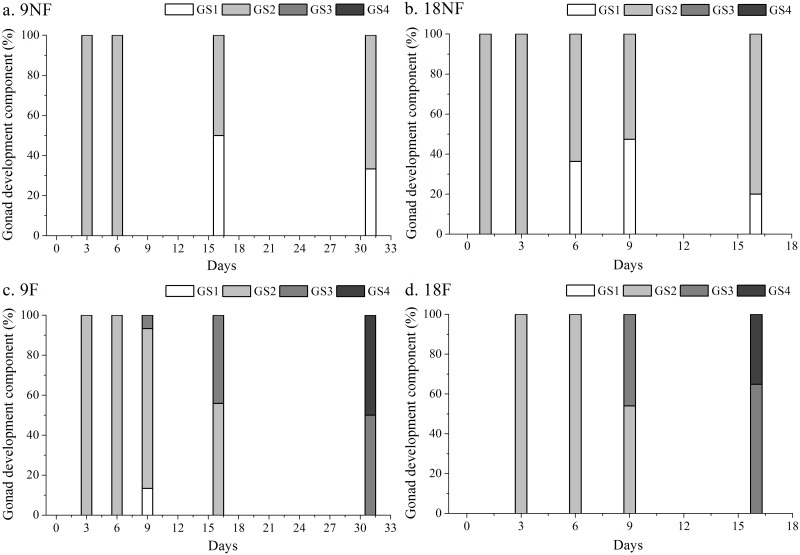
Time series of gonad developmental stages in *Calanus sinicus* in different temperatures by food conditions. (a) 9NF (9°C not fed), (b) 18NF (18°C not fed), (c) 9F (9°C fed) and (d) 18F (18°C fed). GS1 to GS4 represent gonad development stages 1–4.

**Fig 8:**
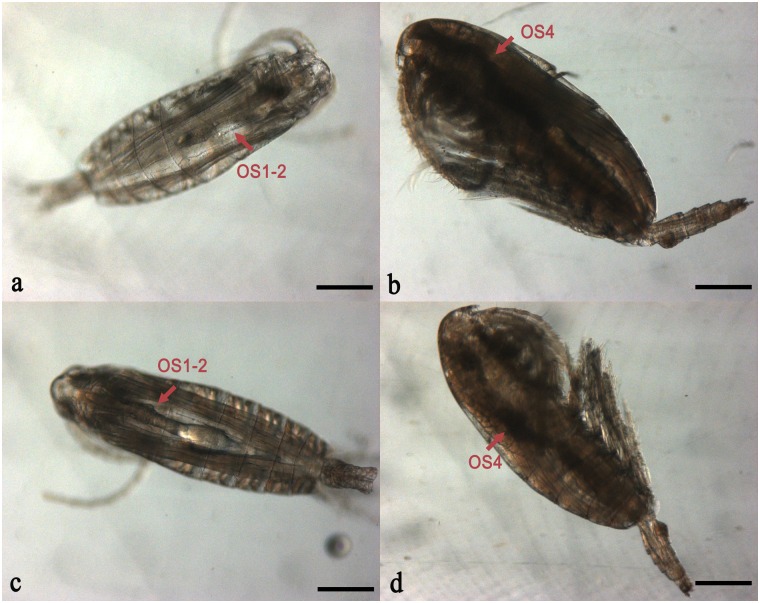
Gonad development states (GS2-GS4) in *Calanus sinicus* at the end of the culture experiment. (a) Group 9NF (9°C not fed), GS2, developing oocytes (OS1 and OS2); (b) Group 9F (9°C fed), GS4, mature oocytes (OS4); (c) Group 18NF (18°C not fed); (d) Group 18F (18°C fed). Scale bar: 400 μm.

Almost all copepodids molted into females except for one male on Day 9 in Group 9F (Fig 4). The gonads of females developed after the final molting, from GS1 to GS4 (Fig 7). Without a food supply during the experiment, all females in Groups 9NF and 18NF developed their gonads into GS1-2 with immature oocytes (oocyte development state 1–2; Fig 8). Reserved lipid could be transformed into phospholipid as both energy and materials for gonad development in *C*. *finmarchicus* at the end of diapause [13, 16, 29, 52]. In the Arctic Ocean, *C*. *glacialis* and *C*. *hyperboreus* could mature and spawn relying only on lipid reserve [14, 53, 54]. However, the reserved lipid could only support the early gonad development (GS1-2) in *C*. *sinicus* after over-summering. In contrast, with food, females eventually matured in Groups 9F (GS4% = 50%) and 18F (GS4% = 35%) on Days 31 and 16, respectively (Fig 7), which is similar to other *Calanus* spp. copepods [12, 29]. Because of food limitation during over-summering period, the gonad of *C*. *sinicus* remains immature and results in null egg production, which increases with the recovery of the food resource [55]. Food bottleneck is also the main factor to suppress the reproduction of three cyclopoid copepods, and induce summer diapause in Lake Schierensee [56]. Therefore, external food supply would be an indispensable energy source for final gonad maturity in *C*. *sinicus*, whereas warmer temperature promoted the development processes.

**Fig 9:**
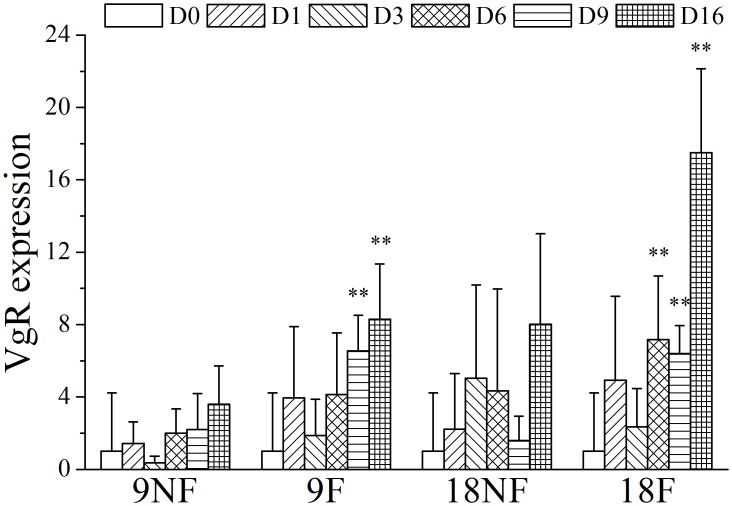
Relative gene expression variation of gene Vitellogenin receptor (VgR) of *Calanus sinicus*. 9NF = 9°C not fed, 9F = 9°C fed, 18NF = 18°C not fed and 18F = 18°C fed. D0 to D16 represent Days 0 to 16. The expression level was normalized to the geometric mean of three housekeeping genes (GAPDH, 16s, EFα1). Error bars show the standard deviation. Significant differences (LSD): *P* < 0.05 (*); *P* < 0.01(**).

When copepods had food, the vitellogenin receptor (VgR) expression increased significantly from Day 9 in Group 9F and Day 6 in Group 18F (Fig 9, ANOVA, *P* < 0.01). Vitellogenesis is a critical part in the gonad maturity process. During the late vitellogenesis process, the vitellogenin receptor mediates the uptake of vitellogenin into the oocytes from the hemolymph when oocytes develop into OS3 in GS3 [57–59], resulting an increased expression of VgR among crustacean and insect species [60–62]. The VgR expression showed an earlier response to food supply than did the occurrence of GS3 gonads on Day 9 in Groups 9F and 18F (Fig 7). The up-regulation of VgR expression occurred 10–22 days before the final maturity. Without food, copepods did not regulate VgR expression significantly (Fig 9) and the gonad remained immature during the experiment. Thus, elevated food might be the external trigger to up-regulate VgR expression and activate final maturity of gonad development.

### Lipid consumption

**Fig 10:**
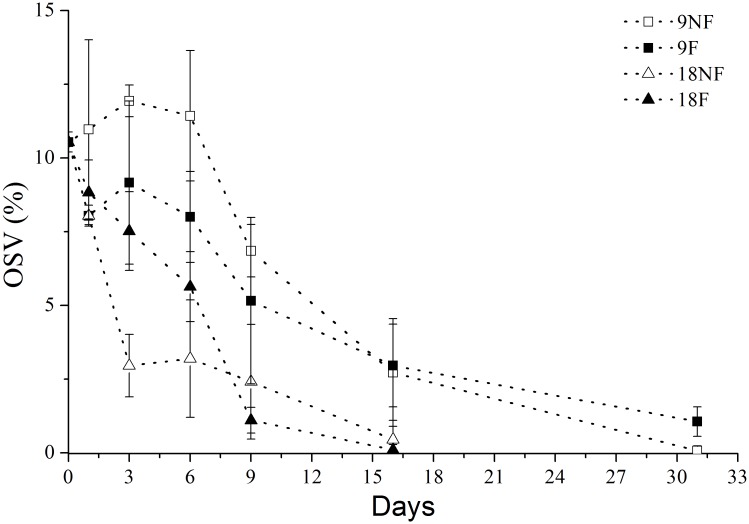
Oil sac proportion (OSV%) of *Calanus sinicus* cultured in different temperatures by food conditions. 9NF = 9°C not fed, 9F = 9°C fed, 18NF = 18°C not fed and 18F = 18°C fed.

The C5s within the YSCWM contained large lipid reserves in their oil sacs (OSV%: 0–30.67%), which is the main energy source for dormant *Calanus* spp. copepods [11, 29]. After the termination of over-summering, the OSV% decreased significantly on Day 1 in Group 18NF and Group 18F (Fig 10, ANOVA, *p* < 0.01), but not until Day 9 in Group 9NF and Group 9F (Fig 10, ANOVA, *p* < 0.01). Elevated temperature accelerated the lipid consumption process after termination of over-summering.

**Fig 11:**
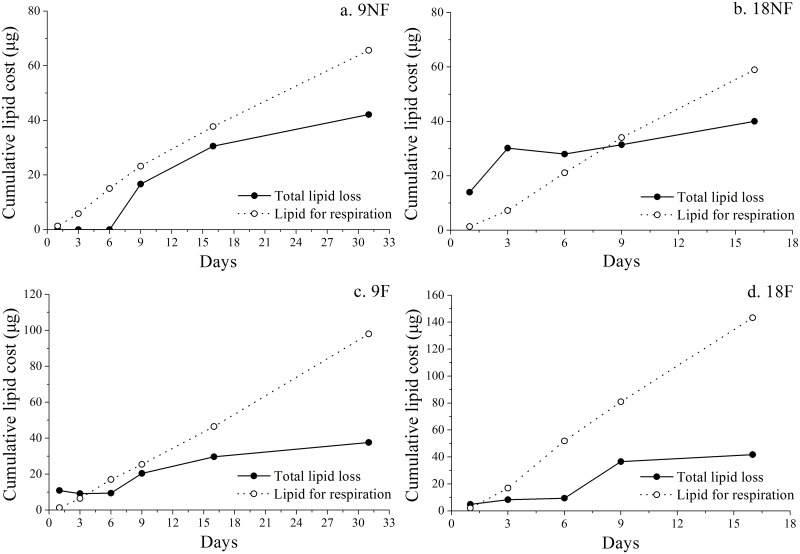
Cumulative lipid costs for respiration and total lipid loss in oil sacs of *Calanus sinicus*. (a) 9NF (9°C not fed), (b) 18NF (18°C not fed), (c) 9F (9°C fed) and (d) 18F (18°C fed).

**Fig 12:**
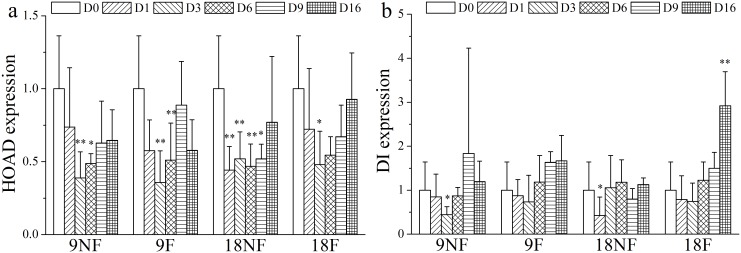
Relative gene expression variation of genes associated with lipid consumption of *Calanus sinicus*. (a) HOAD (hydroxyacyl CoA dehydrogenase) expression. (b) DI (dienoyl-CoA isomerase) expression. 9NF = 9°C not fed, 9F = 9°C fed, 18NF = 18°C not fed and 18F = 18°C fed. D0 to D16 represent Days 0 to 16. The expression level was normalized to the geometric mean of three housekeeping genes (GAPDH, 16s, EFα1). Error bars show the standard deviations. Significant differences (LSD); *P* < 0.05 (*), *P* < 0.01(**).

The total lipid loss and lipid cost for respiration were estimated to reveal the lipid assignment after termination of over-summering (Fig 11). HOAD expression (hydroxyacyl CoA dehydrogenase) was also tested to explore lipid consumption by β oxidization, which is up-regulated when copepods rely only on lipid reserves for energy needs [63, 64]. In the fed groups, the lipid cost for respiration exceeded total lipid loss after Day 3 (Fig 11c & 11d). Meanwhile, HOAD expression levels were down-regulated significantly in Groups 9F and 18F (Fig 12a). Thus, external food would be the main energy source for metabolism and development in these two groups. When unfed, the total lipid loss did not afford the respiration needs all the time in Group 9NF (Fig 11a) and left no extra lipid to support molting. As a result, most of the unfed C5s failed to molt in Group 9NF, even though EcR expression was up-regulated and some C5s began apolysis. With down-regulation of HOAD expression, another energy source (e.g. body carbon) existed to maintain basic metabolic activity in Group 9NF. At higher temperatures, the total lipid loss exceeded the respiration cost in the first eight days of the experiment (Fig 11b). During the period, the copepods molted into adults and the gonads developed in Group 18NF. Only 10–24% lipid loss was utilized for respiration in the first three days, when gonad formation proceeded in the C5 stage [65]. Thus, the major lipid reserve in quiescent *C*. *sinicus* would be used for gonad formation and molting after the termination in Group 18NF, which is similar to *C*. *finmarchicus* [16, 29, 39]. In addition, the HOAD expression was down-regulated and the total lipid loss could not meet the respiration needs in the late period of the experiment, suggesting utilization of other energy sources (e. g. body carbon). Therefore, elevated temperature would accelerate development processes, resulting in a pulse of molting development within a few days after the termination. Thus, elevated temperature or food conditions from the strong vertical mixing of seawater exchange when the YSCWM shrinks in autumn could ensure the success of molting of the over-summering *C*. *sinicus* [10]. The lipid reserve would be utilized both as nutriment and energy for molting and gonad development after the termination, consistent with *C*. *finmarchicus* revived from diapause [16, 29, 39].

The expression pattern of dienoyl-CoA isomerase (DI), an auxiliary enzyme in the β oxidization of polyunsaturated fatty acids (PUFA), differed among the four groups (Fig 12b) [66, 67]. Without food, DI expression decreased significantly on Day 1 in Group 18NF and on Day 3 in Group 9NF (ANOVA, *p* < 0.05). With food, DI expression decreased in the early three days, and then increased slightly in Group 9F and significantly in Group 18F on Day 16 when the gonads became mature (ANOVA, *p* < 0.01). DI expression also showed positive correlation with VgR (Spearman correlation analysis, r = 0.493, *p* < 0.01), indicating increased catabolism of PUFA after gonad maturity, which might be attributed to high PUFA contents in the food supply (*S*. *costatum* and *P*. *micans*) [68].

### Stress tolerance

**Fig 13:**
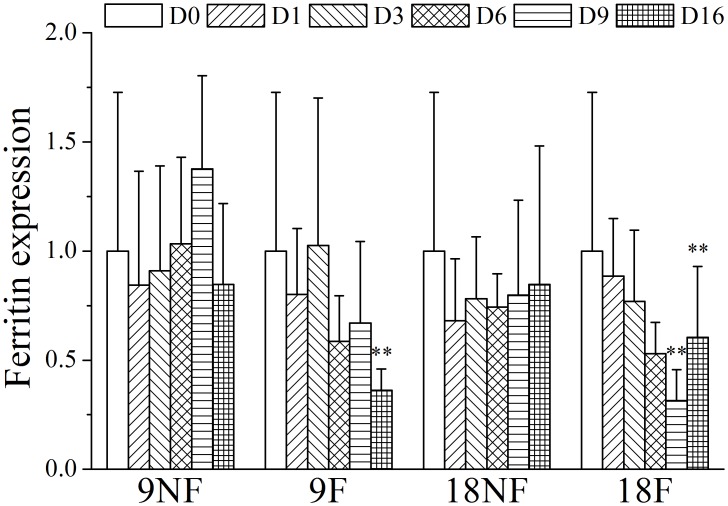
Relative gene expression variation of gene ferritin of *Calanus sinicus*. 9NF = 9°C not fed, 9F = 9°C fed, 18NF = 18°C not fed and 18F = 18°C fed. D0 to D16 represent Days 0 to 16. The expression level was normalized to the geometric mean of three housekeeping genes (GAPDH, 16s, EFα1). Error bars show the standard deviations. Significant differences: LSD, *p* <0.05 (*), *p* <0.01(**).

With the poor food conditions within the YSCWM in summer, *C*. *sinicus* showed high ferritin expression (Fig 13), which is an iron storage protein that could protect the cells and macromolecules from oxidative damage and is believed to suppress development during dormancy [28, 69, 70]. But after termination of over-summering, copepods in the experiment still maintained high expression level of ferritin even during the molting and gonad development processes (Fig 13). When the quiescent eggs of *Acartia tonsa* were recovered from resting stage and hatched, the expression level of ferritin was even 52-times greater than the quiescent status [71]. Thus, the function of high ferritin expression in dormant *Calanus* copepods should be researched further. Ferritin expression was down-regulated significantly only in copepods with food on Day 16 in Group 9F and Day 9 on Group 18F (ANOVA, *p* < 0.01; Fig 13). Thus, ferritin expression might have a sensitive response to limited food, one of the major environmental stresses within the YSCWM. In addition, ferritin expression was strongly correlated with the proportion of C5s (Spearman correlation analysis, r = 0.815, *p* <0.01) and OSV% (Spearman correlation analysis, r = 0.518, *p* < 0.05). Ferritin expression might be related to the development stage in *C*. *sinicus*, whose C5s stage exhibited higher ferritin expression than did females [36].

### Multivariate analysis of gene expression

**Fig 14:**
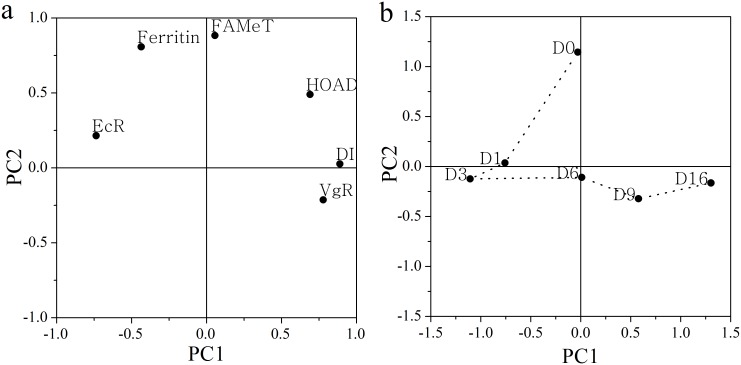
Principal component analysis of the relative gene expression data of *Calanus sinicus* in Group 18F (18°C fed). (a) The loading plot of six genes. (b) Scatterplot of the averages of the first two principal component scores for *C*. *sinicus*. D0 to 16 represent Days 0 to 16.

Principal component analysis (PCA) was conducted to explore the overall regulation patterns of the six genes’ expressions during termination of over-summering and subsequent development in Group 18F, because only those copepods matured within 16 days. The first principal component (PC1) explained 43.45% of the entire variance and was influenced strongly by EcR, VgR, HOAD and DI, representing development and lipid consumption processes. The second principal component (PC2) contributed another 29.38% of the variance, which was mainly affected by FAMet and ferritin (Fig 14a), representing stress tolerance. The average daily values of the PC1 and PC2 scores were shown in Fig 14b. The expression pattern on Day 0 was discriminated from the other days by PC2 (ANOVA, *p* < 0.05) for high expression levels of ferritin and FAMet. The expression patterns on Days 1 and 3 were clustered together for similar scores in PC1 and PC2. The expression pattern on Day 16 was distinct from that on Day 6 based on the PC1 score for significantly up-regulated genes VgR and DI (Fig 14b, ANOVA, *p* < 0.05). But the expression pattern on Day 9 was similar to those on both Days 6 and 16 based on the PC1 and PC2 scores, suggesting a transition phase between them.

**Fig 15:**
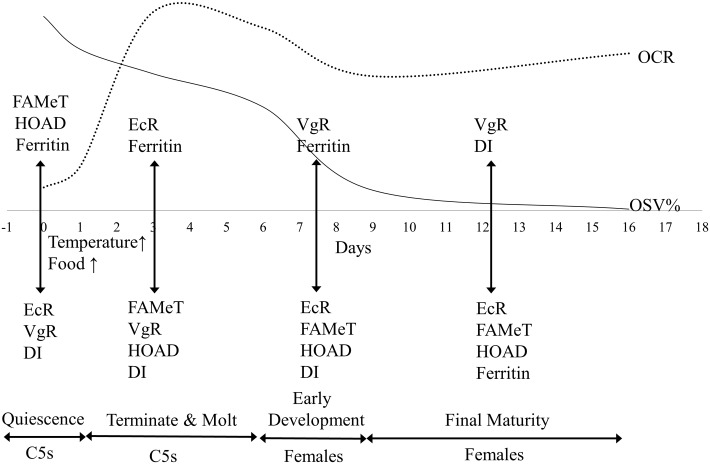
Termination and subsequent development of the over-summering *Calanus sinicus* cultured in Group 18F (18°C fed). The internal processes were divided into four periods: quiescence (Day 0), termination and molting process (Days 1–6), early gonad development (Days 6–9) and final gonad development (Days 9–16). The associated metabolic rates (OCR, dotted line), lipid reserve (OSV%, solid line) and gene expression patterns are shown. See Table 1 for gene abbreviations.

Combined with the physiological and morphological results discussed above, the daily gene expression patterns could be divided into 4 time periods (Fig 15): quiescence (Day 0), termination and molting process (Days 1–6), early gonad development (Days 6–9) and final gonad development (Days 9–16). On Day 0, the copepods would be quiescent, exhibiting suppressed molting process, low metabolic rates and large oil sacs. The quiescent copepods also showed high ferritin, FAMet and HOAD expression and low EcR expression. On Days 1–6, the quiescent C5s were aroused by the elevated temperature and food conditions, exhibiting notably increased metabolic rates. During this period, copepods up-regulated EcR expression and then molted. The lipid reserve in oil sac (OSV%) was reduced slightly with down-regulation of HOAD expression in the first six days. On Days 6–9, newly-molted females began early gonad development (GS 1–2) with up-regulation of VgR and down-regulation of EcR. The lipid reserve was exhausted quickly in this period to support gonad development. On Days 9–16, the gonad matured (GS4) with extremely high expression of VgR. The DI expression was up-regulated, while ferritin expression was down-regulated.

## Conclusion

This study showed the first dynamic profiles of metabolic rates, gene expression and associated physiological processes during the termination of quiescence and subsequent development, as well as their interaction with environmental factors in *C*. *sinicus*. Environmental changes (increased temperature or better food supply) were cues to stimulate the termination of over-summering *C*. *sinicus*. The up-regulation of EcR and down-regulation of HOAD and FAMeT occurred on Day 1 after termination of over-summering, showing a more rapid response to environmental change than did metabolic rates. After the copepodids molted into females, the increased expression of VgR indicated the beginning of gonad maturity. Ferritin was sensitive to the food condition and was down-regulated only when copepods had food. During the whole incubation period, the reserved lipid in the oil sac was depleted to support molting and early development of gonads. The improved temperature and food conditions ensured the success of molting. The external food supply was a trigger to begin the final gonad maturity by up-regulating VgR expression. The food supply also ensured the nutrient and energy needs for the final gonad maturity. Thus, food played an indispensable role in population recruitment when over-summering ended, whereas the elevated temperature accelerated these physiological processes.
